# Analysis of related complications of totally implantable venous access ports in children’s chemotherapy: Single center experience

**DOI:** 10.1097/MD.0000000000029899

**Published:** 2022-07-08

**Authors:** Songze Zhang, Zhangsheng Xiao, Feibiao Yang

**Affiliations:** a Department of General Surgery, The Affiliated People’s Hospital of Ningbo University, Ningbo, Zhejiang, China.

**Keywords:** catheter-related blood stream infection, children, complications, totally implantable venous access port

## Abstract

Totally implantable venous access port (TIVAP) has become an important infusion channel for children who need chemotherapy. With the popularization of TIVAP, its related complications have gradually received clinical attention. However, there are few studies on the complications of TIVAP in children. Therefore, this study intends to analyze the risk factors of complications in children’s infusion port, so as to provide basis for guiding clinical prevention and intervention.

This paper retrospectively analyzed 182 children who received TIVAP implantation in our hospital from January 2018 to January 2021. According to the demographic data, basic disease status and operation related data obtained through Hospital Information System and manual follow-up, the complications and related influencing factors after implantation and implantation were summarized and analyzed. SPSS software was used to analyze the influencing factors between the complication group and the control group.

There were 182 cases of children implanted in intravenous infusion port, of which 71 cases had complications, infection was the most common complication in 50 cases, followed by catheter blockage in 23 cases. Among the infection factors, catheter-related blood stream infection accounted for the highest proportion in 31 cases (17.0%), and *Staphylococcus epidermidis* was the most common pathogen. A total of 19 cases were pulled out early, and the unplanned pullout rate of catheter-related blood stream infection was the highest. In the analysis of influencing factors, age had significant differences in catheter-related infection, all complications and no complications (*P* < .05).

The overall incidence of complications in the use of TIVAP in children with chemotherapy is high, and infection is the most common complication, among which catheter-related blood stream infection is the most common cause of unplanned pullout. Lower age may be associated with a higher incidence of complications.

## 1. Introduction

Totally implantable venous access port (TIVAP) is widely used in clinic because of its advantages of long-term retention, easy management, and low infection rate.^[1]^ Although TIVAP has higher acceptance than traditional peripheral intravenous chemotherapy, infusion port-related complications still occur in the process of long-term use. The occurrence of complications will prolong the length of stay, increase additional hospitalization expenses, and lead to additional pain for children.^[2,3]^ The increase of unplanned port removal will inevitably lead to secondary puncture, increase the risk of vascular injury and recurrent complications, and increase the medical burden.^[4]^ At present, the time of TIVAP implantation in developing countries is still short, and there are few studies specifically aimed at the related complications of children who need long-term chemotherapy in TIVAP. Based on the clinical data of 182 children who undergo TIVAP implantation in our center, this study evaluated the related complications and analyzed the risk factors, hoping to improve the awareness of early clinical prevention and treatment.

## 2. Materials and Methods

### 2.1. Patients

The data of 182 children who received TIVAP implantation and required long-term chemotherapy in the People’s Hospital Affiliated to Ningbo University from January 2018 to January 2021 were collected. Age 0 to 10 years old, the primary diseases include solid organ tumors, leukemia, lymphoma, and so on. First inclusion criteria: (1) Have complete clinical data; (2) Children and their parents can cooperate to complete the examination and follow-up; Second exclusion criteria: (1) Those with systemic infection; (2) There are secondary immune deficiencies or those that affect this study. Other organ dysfunction; (3) Other nontumor chemotherapy with infusion port placement (such as pretransplantation pretreatment). This study was approved by the Human Research Ethics Committee.

### 2.2. Surgical technique of TIVAP placement

**Figure 1.**
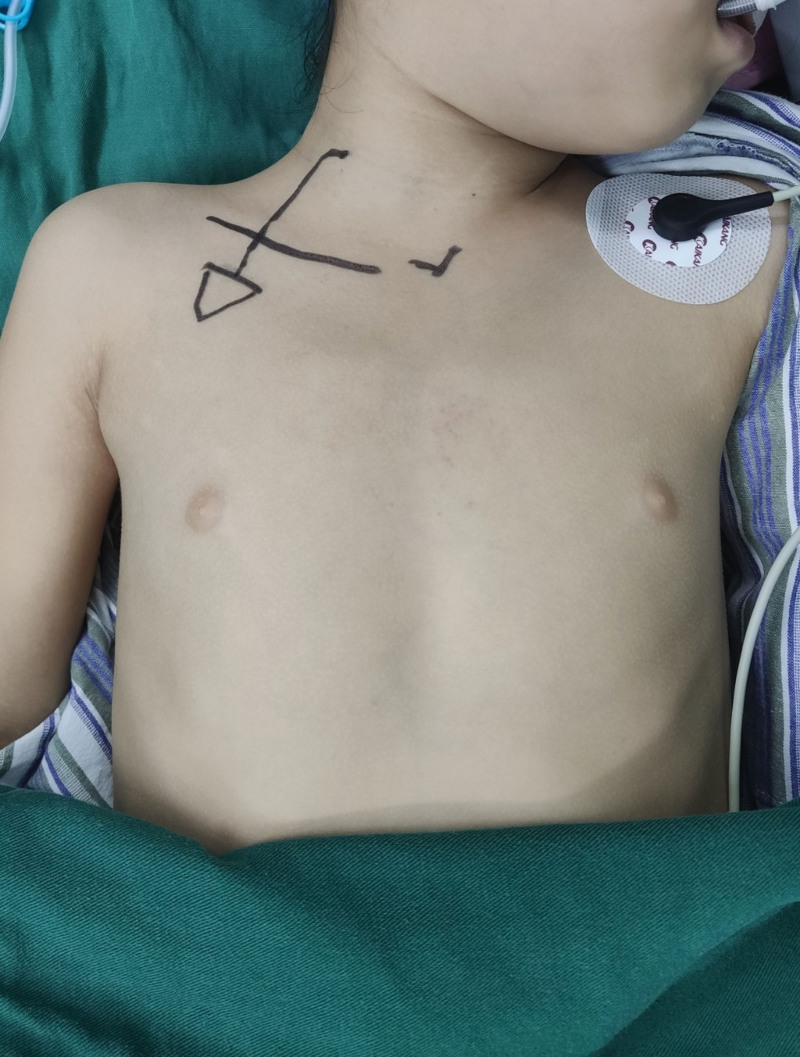
Preoperative body surface positioning of the puncture line.

**Figure 2.**
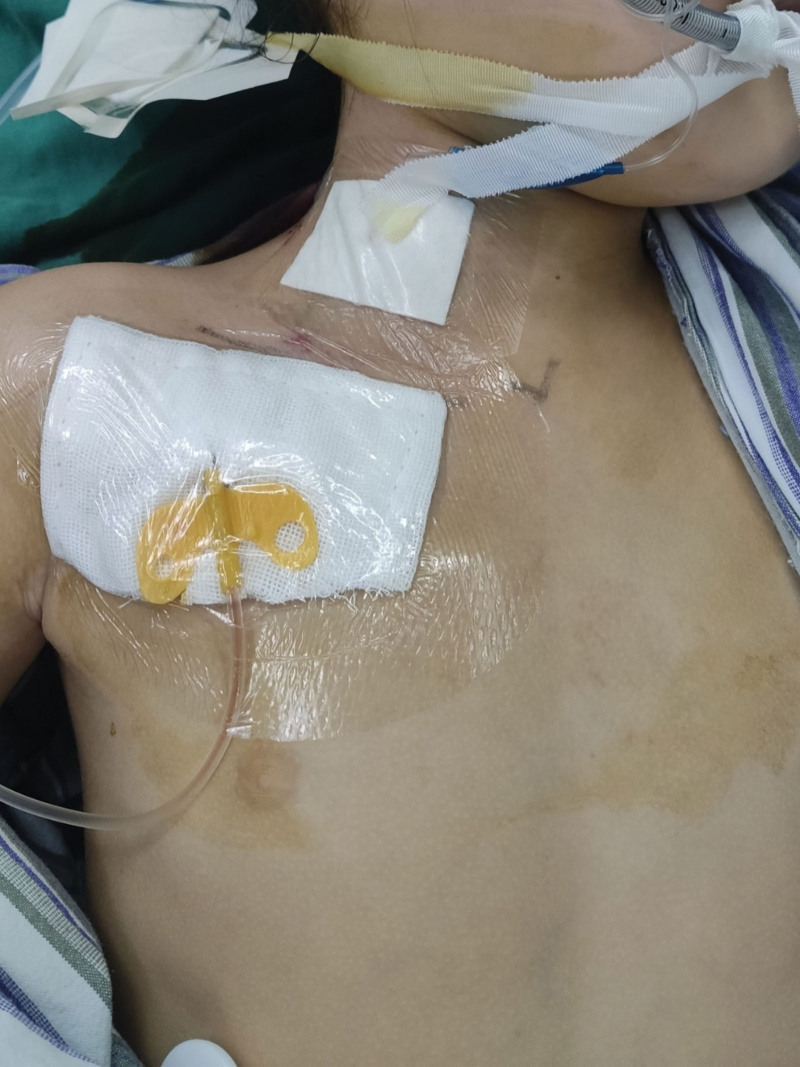
State after suturing the incision.

All children were under general anesthesia in the operating room. We do not use antibiotics prophylactically before and during surgery unless the patient has a definite infection. The venous access port device is made by Bard company of the United States. It is a single lumen catheter with guidewire and peel-away sheath. We used ultrasonography (USG)-guided technique for surgery. We choose the internal jugular vein as the puncture vessel. The patient is in a supine position with the neck tilted to the opposite side and the shoulder slightly raised by support. First, we mark the body surface (Fig. 1), make a 2–3 cm transverse incision 2 cm on the anterolateral aspect of the chest. A subcutaneous pocket is created on the inferior aspect to accommodate the port. The neck marking point is the ideal place for the puncture point of internal intravenous vein. A subcutaneous tunnel is created connecting both the incisions, and the catheter is guided along that tunnel. Then, USG-guided venipuncture is made though which the guidewire is inserted. The tract is dilated with serial dilators. Gently rotate along the guide wire, insert the stripping sheath, and remove the stylet of the sheath. At this time, the pressure blood may gush out, the operator can block the sheath with his thumb to block the bleeding, and then quickly insert the catheter. During the operation, the bedside X-ray is used to determine the appropriate position of the catheter head, the excess length is cutoff, the catheter is connected to the port and placed in the reserved subcutaneous pocket. We generally do not fix the port to the chest. The patency and reflux are ensured by injecting heparin diluted with isotonic (10 U/mL)^[5]^ and suction. At last, we suture the incision of neck and chest incision (Fig. 2). After the operation, TIVAP was maintained according to the consensus of Chinese experts.^[4]^ We used 10% povidone iodine to disinfect the skin. The catheter was flushed and sealed by injecting heparin diluted with isotonic (10 U/mL). The single use of the nondestructive needle will not exceed 1 week. After removing the noninvasive needle, the transparent dressing is required to be kept for >24 hours. The catheter was flushed once every 4 weeks during the infusion interval.^[4]^

### 2.3. Data collection

We obtained the general clinical data of patients through hospital information system, including gender, age, tumor type, chemotherapy information, surgical result, time from completion of TIVAP to first use, absolute number of neutrophils, and the operation time. These patients were followed up until the end of chemotherapy or unplanned port removal, and their complications were recorded, including infection factors and noninfectious factors.

### 2.4. Statistical analysis

Data were analyzed using the Statistical Product and Service Solutions (SPSS) package (SPSS 13.0 for Macintosh). We grouped the patients according to whether there were complications related to the infusion port during the follow-up. T-test or χ^2^ tests as used to analyze the continuous variables of the 2 groups, such as gender, age, tumor type, preoperative chemotherapy, and so on. The measurement data are expressed in median (m), and the comparison between group adopts t-test. The counting data were in cases (%), and the comparison between groups was used or Fisher exact probability method, and logistic-regression analysis was carried out for meaningful influencing factors. The difference was considered to be statistically significant (*P* < .05).

## 3. Results

### 3.1. General information

A total of 182 children were included in this study, including 99 males and 83 females, with a median age of 45 (21–216) months. The majority of the patients in this group were hematological malignancies (123 cases, 67.6%). The first chemotherapy of 89 children was carried out after TIVAP implantation. The median time from TIVAP implantation to the beginning of chemotherapy was 3 (0–28) days. Ninety-three children had received chemotherapy by other channels before TIVAP implantation, and the median time from TIVAP implantation to the beginning of chemotherapy was 10 (1–39) days.

### 3.2. Complications

In this group, 71 patients (71/182, 39%) had complications, with a total of 83 cases, of which infection related complications were the most common, with a total of 50 cases (50/83, 60.2%). *Staphylococcus epidermidis* catheter-related bloodstream infection was the most common pathogen of catheter-related bloodstream infection. Among all patients with complications, 19 patients removed TIVAP due to complications, of which 16 cases removed TIVAP due to concurrent infection (10 cases were catheter-related blood stream infection [CRBSI]; 6 cases were local infection), and 3 cases were due to catheter obstruction (Table 1).

**Table 1:** Port-related complications and removal rate of complications in 182 cases

Complication	Number of cases occurred (n = 83)	underwent port removal (n/%) (n = 19)
Infection factors		
Skin infections around the port	31 (17.0%)	6 (19.4%)
Catheter-related bloodstream infection	19 (10.4%)	10 (52.6%)
Noninfectious factors		
Catheter blockage	23 (12.6%)	3 (13.0%)
Bleeding or hematoma	7 (3.8%)	0 (0)
Drug extravasation	2 (1.1%)	0 (0)
Turnover of port	1 (0.5%)	0 (0)

### 3.3. Analysis of risk factors for complications

There was significant difference in age between 71 patients with infusion port–related complications and 111 patients without complications (*P* = .02). There were no significant differences in gender, tumor type, preoperative chemotherapy, first puncture failure, time from completion of TIVAP to first use, absolute number of neutrophils and operation time between the case group and the control group (*P* > .05) (Table 2). Multivariate logistic-regression analysis showed that age had statistical significance for the occurrence of complications (OR 1.545; CI, 0.945-2.236; *P* = .024) (Table 3).

**Table 2:** Analysis of risk factors for complications (n = 182)

Categorical variables	Complication group (n = 71)	Control group (n = 111)	Statistical value	*P*
Gender			χ^2^ = 0.639	0.423
Male	36	63		
Female	35	48		
Tumor type			χ^2^ = 0.408	0.815
Leukemia	48	75		
Lymphoma	3	7		
Malignant solid tumor	20	29		
Received chemotherapy before operation			χ^2^ = 2.576	0.108
No	40	49		
Yes	31	62		
The first puncture failure			χ^2^ = 0.033	0.855
No	68	107		
Yes	3	4		
Age (mo)	41.84 ± 33.56	56.89 ± 46.85	t = 2.347	0.020
Time from completion of TIVAP to first use (d)	9.15 ± 12.54	12.85 ± 26.12	t = 1.113	0.267
Absolute number of neutrophils (10^9^/L)	2.84 ± 3.58	2.69 ± 2.32	t = 0.343	0.731
Operation time (min)	45.21 ± 19.58	48.59 ± 20.64	t = 1.099	0.273

**Table 3:** Multivariate logistic-regression analysis of risk factors for complications (n = 182)

Variable	B	SE	Wald	*P*	OR	95% CI
Age (mo)	–0.052	0.022	5.021	0.024	1.545	0.945-2.236

CI = confidence intervals, OR = odds ratio.

There were significant differences in age (*P* = .015) and absolute number of neutrophils (*P* = .006) between 19 patients with CRBSI and 111 patients without complications. There were no significant differences in gender, tumor type, preoperative chemotherapy, first puncture failure, time from completion of TIVAP to first use and operation time between the case group and the control group (*P* > .05) (Table 4). The results showed that low-age had statistical significance for CRBSI (OR 1.179; CI 0.243-2.077; *P* = .035), and the number of neutrophils was not significant (OR 0.964; CI 0.168-2.254; *P* = .054) (Table 5).

**Table 4:** Analysis of risk factors for catheter-related bloodstream infection (n = 130)

Categorical variables	CRBSI group (n = 19)	Control group (n = 111)	Statistical value	*P*
Gender			χ^2^ = 0008	0.926
Male	11	63		
Female	8	48		
Tumor type			χ^2^ = 1.379	0.501
Leukemia	13	75		
Lymphoma	0	7		
Malignant solid tumor	6	29		
Received chemotherapy before operation			χ^2^ = 1.049	0.305
No	6	49		
Yes	13	62		
The first puncture failure			χ^2^ = 0543	0.819
No	18	107		
Yes	1	4		
Age (mo)	26.52 ± 25.94	56.89 ± 46.85	t = 2.748	0.006
Time from completion of TIVAP to first use (d)	8.84 ± 11.25	12.85 ± 26.12	t = 0.657	0.512
Absolute number of neutrophils (×10^9^/L)	3.98 ± 3.65	2.69 ± 2.32	t = 2.038	0.043
Operation time (min)	44.74 ± 18.46	48.59 ± 20.64	t = 0.762	0.447

TIVAP = totally implantable venous access port.

**Table 5:** Multivariate logistic-regression analysis of risk factors for catheter-related bloodstream infection (n = 130)

Variable	B	SE	Wald	*P*	OR	95% CI
Age (mo)	–0.027	0.015	4.256	0.035	1.179	0.243-2.077
Absolute number of neutrophils (×10^9^/L)	0.168	0.078	3.754	0.054	0.964	0.168-2.254

CI = confidence intervals, OR = odds ratio.

## 4. Discussion

In this study, the incidence of complications related to the use of TIVAP in children’s chemotherapy is 39%, which is higher than 14.7% to 33% reported in foreign children’s studies,^[6,7]^ which may be related to the short time of using TIVAP in our hospital. Infectious complications are the most common. TIVAP-related infection generally comes from the skin infection at the puncture point, and microorganisms migrate into the catheter along the port; or blood stream infection from other distant parts of the body. There are also very few possible sources of pollution caused by infusion liquid.^[8]^ The results of a meta-analysis^[9]^ showed that infection prevention strategies and the education and training of professional nursing staff are the most effective means to prevent infection. The incidence of TIVAP-related infection complications in this group was 27.4%, higher than 6.5% to 17.9% reported in the literature,^[7,10]^ which may be related to the short time of started this operation and lack of long-term TIVAP nursing experience in our hospital. A more recent study in the literature^[7,10]^ reported the lower incidence rate of infectious complications, suggesting that a large number of cases, experienced surgical procedures and maintenance procedures, and standardized care reduced the incidence of infection. In previous literature reports, coagulase-negative staphylococcus (*Staphylococcus epidermidis*, Human staphylococcus, and *Staphylococcus saprophytes*) are the most common infectious microorganisms, among which *Staphylococcus epidermidis* is the most common.^[8,11]^ Similar results were observed in this study. Coagulase-negative staphylococcus is a common skin microorganism, suggesting that the infection may come from the puncture site. This is consistent with the pathogenesis of infection complications. In this study, intravenous anti infection therapy was used after catheter infection, and the effective rate was 75%.

There are many reasons for TIVAP catheter blockage, which can be divided into catheter factors, such as catheter discount, compression or improper end position, catheter tip sticking to the vessel wall or catheter displacement, and noncatheter factors, such as precipitation caused by parenteral nutrition infusion, blood products, and drug interaction during use. Mechanical blockage is often caused by catheter discount or compression. Pinch off syndrome is the most serious mechanical blockage, which is common in subclavian vein catheterization. The catheter is compressed between the clavicle and the first rib for a long time, resulting in the fragmentation of the catheter wall, the debris falling off and moving to the central vein, right atrium, right ventricle, or pulmonary artery. It is a rare but serious complication.^[12]^ In this study, internal jugular vein puncture was used, so no such complication was found. Endothelial injury, hypercoagulable state of malignant tumors, and chemotherapy itself are all factors leading to thrombosis in cancer patients. Thrombosis can occur at the top of the catheter, around, or in the vein. The manifestations of catheter-related thrombosis may be asymptomatic, with or without pain and swelling of the ipsilateral limb.^[13]^ Therefore, the evidence of thrombosis may not be clear clinically, but it is usually the most common cause affecting catheter blockage. In some studies, heparin treatment was compared with urokinase treatment, and it was found that urokinase treatment was significantly associated with lower thrombosis rate.^[14]^ In this study, 91.3% of children with catheter obstruction improved after thrombolytic therapy with heparin or urokinase, suggesting that thrombolytic therapy with heparin and urokinase may reduce the risk of thrombosis. In the past, the incidence of thrombosis was reported to be 1.6% to 7%.^[10]^ No thrombosis cases were found this time, which may be related to the small sample size of this study and the lack of routine regular vascular ultrasound follow-up of patients.

The literature reports that the incidence of drug extravasation is 0.26% to 6%.^[15]^ The severity of lesions caused by extravasation depends on the nature, volume, concentration, and location of extravasation drugs and the contact time required for extravasation drugs to damage surrounding tissues. The prevention of drug extravasation complications should be emphasized. The training of nursing team should be strengthened to prevent serious skin damage caused by chemotherapy drug extravasation and delay tumor treatment. There were 2 cases of drug extravasation in this study. There were no serious consequences after local symptomatic treatment and nursing. The literature reported that the incidence of catheter fracture ranged from 0.4% to 1.8%,^[16]^ which was not found in this study.

In the retrospective analysis of pediatric patients, Hung et al^[17]^ found that low-age patients have a higher risk of infection after TIVAP implantation. The results of logistic-regression analysis in this study suggest that the lower the age, the higher the risk of complications, which is consistent with the reported results and may be related to the nursing difficulties of low-age patients. Young children are prone to resistance or a large swing of the upper limbs during each dressing change. The transparent dressing is more likely to fall off than adults because of their active nature, these may lead to complications such as infection. It has been reported^[18]^ that delaying the use of catheter can reduce the risk of infection twice. It is suggested that 1 week between the placement of TIVAP and the start of use can reduce the incidence of complications and the risk of unplanned port removal, which may be related to the healing time of incision after implantation. This study did not find the impact of the start time of TIVAP on the incidence of complications. Prospective studies with larger samples may be needed. Some scholars have pointed out that the occurrence of complications has nothing to do with the timing of TIVAP surgery, and some studies have pointed out that the risk of infection in children undergoing TIVAP surgery in the early stage of induction remission treatment of acute lymphoblastic leukemia will increase.^[19]^ It has been reported that neutropenia is a risk factor for catheter-related infection.^[20]^ In this study, it is found that neutrophils in the infection group are higher than those in the control group, and the difference is statistically significant, which is inconsistent with previous reports because the chemotherapy time and intensity of leukemia patients are higher than those of solid tumor patients. Therefore, the degree and duration of potential neutropenia are also higher than those of solid tumors. We found that the incidence of clinical sepsis in leukemia group is very high. However, since blood was not collected from all patients with sepsis for culture, the infection rate of catheter-related blood influenza in leukemia group may be underestimated.

## 5. Conclusion

TIVAP, as a device that can easily provide long-term venous access, is safe for pediatric malignant tumors. Special attention is still needed in the process of surgical placement and operation. Infection complications are the most common complications, and CRBSI is the most common cause of unplanned pullout. Young age may be a risk factor for CRBSI and all complications. Surgeons and nursing teams need professional training, hand hygiene needs to be emphasized in the dressing change of low-age children.

### 5.1. Limitations

This paper is a retrospective study. The record of complication outcome comes from the case system data, which may have defects and have a certain impact on the collection of results. Our center started this operation late and lack of long-term TIVAP nursing experience. This may lead to an overestimation of complications. The sample size of this paper is small, and some infusion ports are still in the period of infusion with port, which may have a certain impact on the results.

## Acknowledgements

We acknowledge the work of the nurses and doctors of The Affiliated People’s Hospital of Ningbo University involved in the surgeries.

## Author contributions

Conceptualization: Songze Zhang.

Data curation: Songze Zhang, Zhangsheng Xiao.

Formal analysis: Songze Zhang.

Investigation: Songze Zhang, Zhangsheng Xiao, Feibiao Yang.

Methodology: Songze Zhang, Zhangsheng Xiao.

Project administration: Zhangsheng Xiao.

Resources: Zhangsheng Xiao,Feibiao Yang.

Software: Songze Zhang.

Supervision: Songze Zhang.

Writing – original draft: Songze Zhang.

Writing – review & editing: Songze Zhang.
